# Optimization of the Fermentation Process and Sensory Evaluation of Wild Loquat (*Uapaca kirkiana*) Fruit Wine

**DOI:** 10.1155/ijfo/1716582

**Published:** 2025-09-10

**Authors:** Tatenda A. Mutangadura, Shepherd Manhokwe, Talknice Z. Jombo, Desmond T. Mugadza

**Affiliations:** ^1^ Department of Food Science and Nutrition, Midlands State University, Gweru, Zimbabwe, msu.ac.zw

**Keywords:** fermentation, fruit wine, optimisation, RSM, wild loquat (*Uapaca kirkiana*)

## Abstract

*Uapaca kirkiana* (family Euphorbiaceae), known as wild loquat, is a tropical indigenous fruit tree. The fleshy pulp of the *U. kirkiana* fruit is eaten fresh or processed into a variety of products, including juices, squashes, wines, sweet beer, porridge, jams and cakes. However, the commercialisation of the fruit wines still remains a challenge due to a lack of consistency in product quality. The purpose of this study was to use response surface methodology (RSM) to optimise the fermentation of wild loquat (*U. kirkiana*) fruit wine. A study to investigate the relative contributions of two predictor factors (Brix value and yeast quantity) to the quality of wine was conducted using RSM. Design Expert 7 was used for a central composite design (CCD). Thirteen (13) runs of wine were produced as outlined in the CCD for the independent variables. The responses measured included alcohol by volume (ABV percentage), density, real extract (Er), original extract, Plato, real degree of fermentation (RDF), pH, calories and colour. Overall, Brix had the greatest influence on responses, showing substantial correlations whether they were positive or negative. In contrast, yeast and responses tended to have weaker correlations, with the exception of pH, where the converse was true for both independent components. The optimum values of Brix and yeast for the fermentation of *U. kirkiana* wine were found to be 24.44°Bx and 1 g, respectively. The predicted values for the responses are 11.83% (ABV), 1.00539 g/cm^3^ (density), 75% (RDF), 5.53 EBC (European Brewery Convention) colour, 350.998 kJ/100 mL or 87 kcal and pH of 3.5. The developed models could predict the quality of wine developed from wild loquat fruit. It was observed that wine samples with high Brix and moderate yeast quantities had more intense flavour characteristics. In contrast, wine samples with extreme (high and low) initial yeast levels exhibited strong off odours and sourness.

## 1. Introduction

Wine is a completely or partially fermented juice of the grape, but fruits other than grapes have also been utilised for the production of wines. An estimated 260‐million hectolitres of wine was produced in 2019 [1]. Although the most common type of wine is grape wine (red and white), fruit wines are gradually gaining momentum around the world as a way to make use of underutilised fruits, reduce postharvest losses and impart the health benefits of wine. Fruit wines have proved to be an excellent dietary source of antioxidants, phytonutrients and minerals [2]. Ethyl alcohol, sugar, acids, stronger alcohols, tannins, aldehydes, esters, amino acids, minerals, vitamins, anthocyanins, and flavouring substances are all common components in wine [3, 4]. Wines retain the majority of the nutrients included in the original fruit juice because they are fermented from fruit and are not distilled [5].

Combined with a pleasant taste, fruit wines have been shown to be a source of a variety of phenolic compounds with antioxidant activity [6]. The most prevalent polyphenols, flavonoids, are found in abundance in wine made from fruits. In the source materials (fruits), many polyphenols and other bioactive chemicals are bound to insoluble plant molecules. The fermentation process frees up many of these bioactive substances into an aqueous ethanol solution, making them more physiologically available for human consumption [2]. Fruit wine has a wide range of health benefits on human health. It has been used as a therapeutic agent and is highly acclaimed for its medicinal or therapeutic value [7]. This is attributed to antioxidant polyphenolics, thereby providing an effective means of preventing and treating free radical mediated diseases like diabetes mellitus, neurodegenerative disorders (Parkinson’s disease [PD], Alzheimer’s disease [AD] and multiple sclerosis [MS]) and cardiovascular diseases (atherosclerosis and hypertension) [8].

There are two main differences in the production of wine made from grapes and fruit wines. First, the juice extraction for fruits is usually harder, and second, the sugar content of fruits is usually lower and the acidity level higher than that of grapes [9]. The yeast (*Saccharomyces cerevisiae* var. *ellipsoideus)* is the microorganism on whose activity the production of wine or any other alcoholic beverage depends [10–13]. The nutritive value of wine is also increased due to the release of amino acids and other nutrients from yeast during fermentation. In a fermentation process, it has been estimated that starting with 22%–24% sugars, 95% of the sugars is converted into ethanol and carbon dioxide, 1% is converted to cellular biomass and the remaining 4% is converted to other end products [3]. *Uapaca kirkiana* is highly regarded for its edible fruit and is used to prepare various food products [14]. The juices and jams made from wild loquat are a rich source of zinc, copper and phosphorus, meaning that the consumption of this fruit in its raw or minimally processed form adds to the diet by providing minerals important for metabolism and bone formation [15]. Wild loquat fruit can produce a pulp yield of 28.6% of total fruit weight. In a study by Saka et al. [15], the whole fruit was found to contain 86.5% total carbohydrate, 8.4% fibre, 1.1% fat, 1.8% crude protein and 27.4% dry matter. It was also noted that the specific sugars in *U. kirkiana* fruit are 41 g/100 g glucose, 27 g/100 g fructose, 15 g/100 g sucrose, 2 g/100 g xylose and traces of galactose, raffinose and ribose. *U. kirkiana* fruit can be used as an important source of iron, with its iron content (11.8 mg/100 g EP) being higher than that in most indigenous fruits [16]. Wild loquat was also found to be an excellent source of zinc (1.3 mg/100 g), magnesium (39 mg/100 g), calcium (17 mg/100 g) and phosphorus 915 mg/100 g). However, it is important to note that not all these minerals are available when the fruit is consumed raw and become more available after processes such as fermentation. Fermentation is known to increase the phenolic content of fruit juice products by increasing extraction from the skins and therefore may increase the antioxidant capacity [17] and potential for health benefits [3]. The *U. kirkiana* fruit wine contributes to the nutritional well‐being of individuals. The fruit contains vital nutrients and essential vitamins that are important for coronary ailments or related diseases. Thus, the fruit can be processed into functional foods like fruit wines which can be commercialised.

Response surface methodology (RSM) is a collection of statistical and mathematical techniques useful for developing, improving and optimising processes [18]. RSM has important applications in the design, development and formulation of new products, as well as in the improvement of existing product designs. RSM is mainly used in situations where several input variables can potentially influence performance measures or quality characteristics of the product or process. RSM creates a link between responses and control variables and guesses the response values of the control variables within a specific range [19, 20]. The effect of fermentation conditions on the total alcohol content of wines was investigated by a number of researchers, and the optimum fermentation conditions were determined using RSM ([20–23]. However, the application of RSM in optimisation of indigenous fruit wines has been limited. This study is aimed at optimising the alcoholic fermentation of *U. kirkiana* fruit wine using RSM and central composite design (CCD). In the field of winemaking, the use of mathematical modelling for fermentation optimisation is still limited due to the complexity of the desired quality attributes, which include not only fruit wine quantitative properties but also, with major importance, qualitative properties. A systematic approach was carried out focusing on mathematical modelling and optimisation of fermentation parameters to maximise ethanol production. Fruit wines from *U. kirkiana* were characterised to establish chemical–sensorial correlations and assess acceptability.

## 2. Methodology

### 2.1. Source of Materials

Wild loquat fruits were collected from Shurugwi, Zimbabwe, in November before the onset of the rainy season. Brewer’s yeast was procured from a local yeast manufacturing company, and the sugar (sucrose) was purchased from a local supermarket. All the wild loquat fruit samples were packed in polypropene carrier bags and stored under dry conditions at room temperature (25°C) for further use. Reagents and apparatus used for analysis were obtained from the Food Chemistry Laboratory, Department of Food Science and Nutrition, Midlands State University, Zimbabwe.

### 2.2. Fruit Juice Extraction

The wild loquat fruits were sorted to remove the unripe, overripe and damaged ones. The fruits were then washed thoroughly to remove any dirt. Once clean, they were crushed and mixed with distilled water. The fruit pulp was mixed with distilled water (1:1 *w*/*v*) [24]. The result was a fruit skin, pulp and seeds mix which was sieved using a colander, kitchen sieve and a cheese cloth in succession to produce wild loquat juice (must). Filtration was done using muslin cloth, sieve and syphon tubes sterilised by 70% alcohol. The fruit must was treated with potassium metabisulfite (0.2 ppm) to inactivate wild yeasts and prepare for *S. cerevisiae* fermentation.

### 2.3. Experimental Design

The juice degrees Brix value was measured using a handheld refractometer (Atago, Japan) to establish initial sugar concentration. A study to investigate the relative contributions of two predictor factors (degrees Brix value and yeast quantity) to the quality of wine was conducted using RSM. Design Expert 7 (Stat‐Ease Corporation, Minneapolis, United States) was used to construct a CCD. Thirteen (13) runs of wine were produced as outlined in the CCD for the variables (Table 1). The range of values for the independent variables was defined by the operational values used in the commercial production of wine. The must was mixed with a sucrose solution (1:1 *v*/*v*) to adjust the sugar concentration to between 15°Bx and 25°Bx. The Brix values in 1 L fermentation vessels were then adjusted for each individual sample to meet the stipulated Brix in the design. The response variables for the fermentation process included ABV percentage (alcohol by volume), density, Er (real extract), Plato (original extract), RDF (real degree of fermentation), pH, calories and colour.

**Table 1:** Design summary for the independent variables in central composite design using Design Expert 7.0.

Study type	Response surface						
Initial design	Central composite						
Design model	Quadratic						
Runs	13						
Blocks	No blocks						
**Factor**	**Name**	**Units**	**Type**	**Low actual**	**High actual**	**Low coded**	**High coded**
A	Brix	°Bx	Numeric	15.00	25.00	−1.000	1.000
B	Yeast	g	Numeric	1.00	8.00	−1.000	1.000

### 2.4. Fermentation

Plastic containers (1 L) with airtight lids were used as fermentation vessels. The containers were kept in a cool dark place at 24°C. The wine was fermented for 6 weeks. The fermentation is complete when the Brix level is stable [6]. To ensure no further fermentation occurred, the wine was sonicated (KD‐250B, China) for 10 min [23]. Two days after sonication, the wines’ pH and degrees Brix were tested once more and had not changed. This marked the completion of fermentation. The wines were then moved from the fermentation vessels to inert airtight glass jars. The wines were then clarified using gelatin and kept in a cool environment, monitored daily. After 2 weeks, sediments had formed at the bottom of the wine. The clear wine was then syphoned out using a peristaltic pump (Cole‐Parmer, Masterflex 7554‐90, United States) for storage and further analysis.

### 2.5. Analytical Methods

A Benchtop pH metre (Milwauke Mi150, Romania) was used to determine the pH of the *Parinari curatellifolia* wine samples using the AOAC [25] procedure. The soluble sugars concentration was determined with a refractometer (S‐DT‐016, Japan). One drop of fruit juice and wine was applied to the refractometer, and readings in degrees Brix were obtained and recorded. The Anton Paar DMA 4500M‐EC Beer Alcolyzer at a local brewing plant was used to assess wine quality. The infrared‐based machine measured ABV percentage, density, Er, original extract, Plato, RDF, pH, calories and colour.

### 2.6. Sensory Evaluation

Numerous aspects determine the quality of wines, such as their sensory properties or microelement compositions, which makes them difficult to objectively assess. Conventional profiling (CP) methods, also called conventional descriptive analysis or quantitative descriptive analysis, is a classical method introduced to describe the sensory characteristics of products based on intensity ratings of descriptors. A semitrained panel of 13 people was used. The panel was recruited based on interest, availability and previous performance in wine descriptive analysis studies. Each panellist was given a form to fill in when tasting each of the 13 wine samples of what they perceived on wine attributes. Colour, clarity, fruity aroma, ethanol aroma, off odours, sweetness, sourness, body and aftertaste were assessed using 5‐point scales. Panellists were given samples along with score sheets to fill out while tasting the wines. All 13 samples were then analysed, and the results were recorded. XLSTAT 2022 (Addinsoft, Paris, France) was then used to analyse the results. Principal component analysis (PCA) was used to show the differences between the 13 wines’ flavour and taste characteristics.

## 3. Results

As shown in Table 2, all the wines were found to contain alcohol with amounts ranging from 6.49% to 13.76%. The wines’ colour was found to range from 1.3 EBC to 6.01 EBC. The highest degrees Plato recorded was 11.82, and the same run had the highest RDF (90.46%). At an inclusion rate of 8 g/L and Brix value of 25°Bx, the highest calorie content of 402.41 kJ/100 mL was recorded. The pH ranged from 3.48 to 4.35.

**Table 2:** A summary of the central composite design (CCD) for wild loquat wine optimisation using response surface methodology and the responses.

**Wine**	**Block**	**Brix (°Bx)**	**Yeast (g)**	**ABV (%)**	**Plato (°Plato)**	**Density (g/cm** ^**3**^ **)**	**Er (%)**	**RDF (%)**	**Calories (kJ/100 mL)**	**Colour (EBC)**	**pH**
W1	Block 1	15.00	8.00	7.98	19.27	0.9929	1.48	89.18	204.65	4.93	3.94
W2	Block 1	20.00	4.50	11.35	16.08	0.9920	2.29	88.02	293.98	2.98	4.00
W3	Block 1	20.00	4.50	9.66	11.82	0.9909	1.51	90.64	243.42	4.34	3.83
W4	Block 1	27.07	4.50	11.59	18.73	1.0124	7.47	68.67	378.69	5.01	3.62
W5	Block 1	20.00	4.50	10.99	16.58	0.993	2.42	87.07	287.58	3.31	3.60
W6	Block 1	25.00	8.00	13.76	23.30	1.0032	5.82	76.97	402.41	4.56	3.63
W7	Block 1	25.00	1.00	11.55	12.00	1.0100	6.89	70.43	368.74	6.01	3.48
W8	Block 1	12.93	4.50	6.80	19.72	0.9939	1.34	88.66	175.72	2.22	3.64
W9	Block 1	20.00	4.50	9.82	25.27	0.9920	1.82	89.05	251.35	3.84	3.96
W10	Block 1	20.00	4.50	11.85	13.66	0.9911	2.21	88.80	303.96	3.76	4.35
W11	Block 1	20.00	9.45	11.62	19.12	0.9907	2.04	89.39	296.35	3.24	3.59
W12	Block 1	15.00	1.00	6.49	19.92	0.997	2.04	83.01	179.08	1.30	3.66
W13	Block 1	20.00	4.50	11.56	23.84	0.9941	2.90	85.44	305.97	3.86	3.65

ANOVA was used to check the significance of the models developed. The results of the ANOVA, presented in Table 3, indicate that the models developed adequately represented the actual relationships between the independent variables and responses. The *F* values of the models implied that the models for qualities, which were ABV percentage, density, Er, degrees Plato, RDF, colour, calories and pH, were significant. The associated *p* value of the models, which were used to estimate whether *F* was large enough to indicate statistical significance, was all lower than 0.05, indicating that the model developed and the terms were highly significant.

**Table 3:** ANOVA results for each of the response variables.

**Parameter**	**Model**	**Sum of squares**	**Df**	**Mean square**	**F** **value**	**p** **value**	**Model remark**
ABV	Linear	200.93	2	100.47	48.49	< 0.0001	Significant
Density	Quadratic	6.099e − 004	5	1.220e − 004	19.45	0.0006	Significant
Er	Quadratic	52.36	5	10.47	28.34	0.0002	Significant
Plato	Linear	200.93	2	100.47	48.49	< 0.0001	Significant
RDF	Quadratic	605.95	5	121.19	13.99	0.0016	Significant
Colour	Linear	9.39	2	4.70	5.17	0.0287	Significant
Calories	Linear	57330.79	2	28665.39	50.22	< 0.0001	Significant
pH	Linear	0.39	2	0.19	6.52	0.0154	Significant

**Figure 1:**
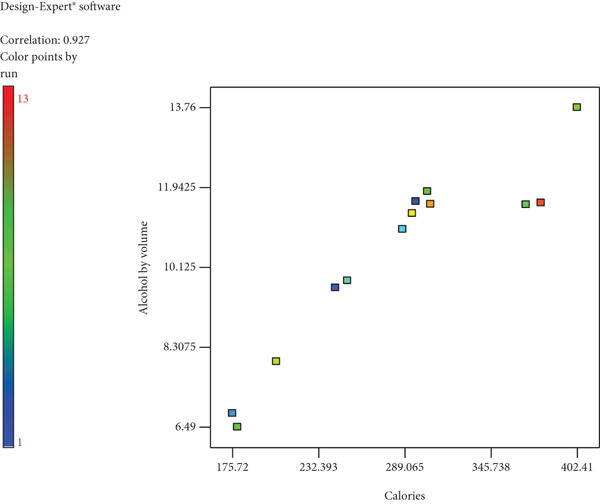
Correlation between alcohol by volume and calories of wild loquat wine.

**Figure 2:**
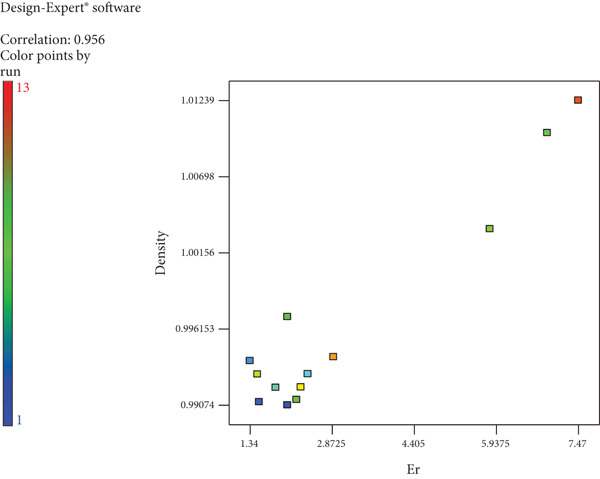
Correlation between density and Er of wild loquat wine.

There is a strong positive correlation (*r* = +0.95) between calories and Brix in wine, as shown in Table 4. The association between yeast and colour is weak. A strong positive correlation (*r* = +0.927) was noted between ABV and calories, as seen in Figure 1. The relationship between Er and density shown in Figure 2 is a strong positive correlation (*r* = +0.956).

**Table 4:** Correlation coefficient (*r*) between independent and response variables during wine making.

**Response variable**	**Brix**	**Yeast**
ABV (%)	+0.838	+0.194
Density (mg/cm^3^)	+0.685	−0.176
Er (%)	+0.852	−0.095
Plato (°Plato)	+0.948	+0.091
RDF (%)	−0.727	+0.201
Colour (EBC)	+0.682	+0.210
Calories (kJ/100 mL)	+0.950	+0.088
pH	−0.211	+0.722

**Figure 3:**
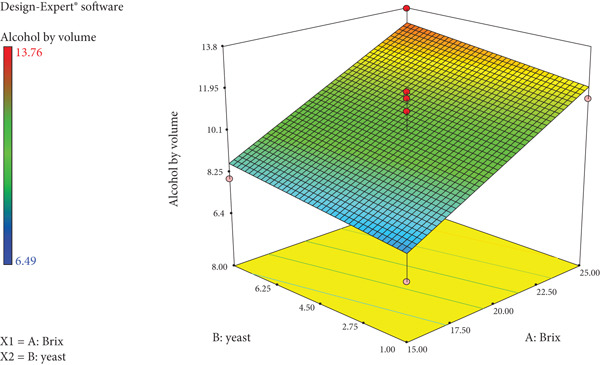
Response surface plot of Brix and yeast on ABV percentage of wild loquat wine.

In order to optimise the parameters and investigate their interactions on the quality of the wine, the visualisation of the predicted models was shown by the surface response plots. 3D response surface plots or contour plots were created using RSM in Design Expert 7.0. Figure 3 shows a linear relationship between Brix and yeast with the ABV percentage of the wines. As the sugar concentration (Brix) and yeast amount increase, so does the ABV percentage of the wine, with the highest alcohol percent (13.76%) noted at 25°Bx and 8 g/L yeast and the lowest noted at 15°Bx and 1 g/L yeast.

**Figure 4:**
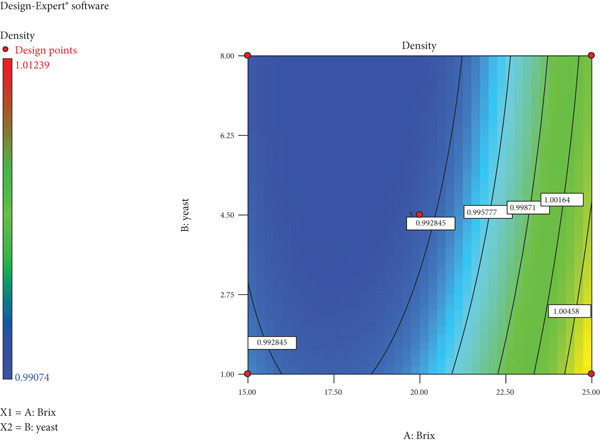
Contour plot for Brix, yeast and density of wild loquat wine.

As evident in Figure 4, the density of mazhanje wine gradually increased with an increase in Brix and a decrease in yeast quantity. A high density (yellow zone) is noted at 25°Bx and a low yeast amount of 1 ppm.

**Figure 5:**
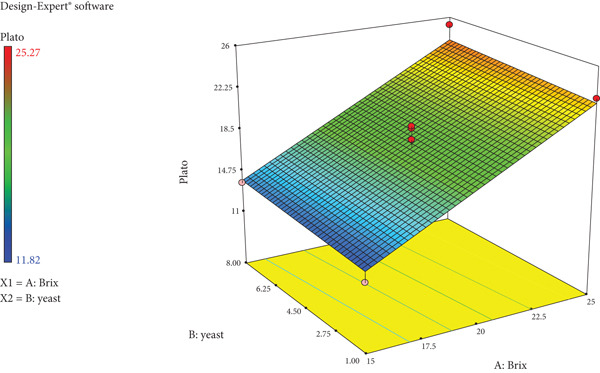
Response surface plot of Brix and yeast on degrees Plato of wild loquat wine.

**Figure 6:**
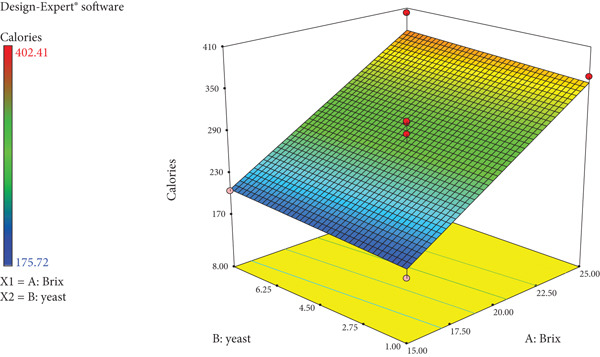
Response surface plot of Brix and yeast on calories of wild loquat wine.

**Figure 7:**
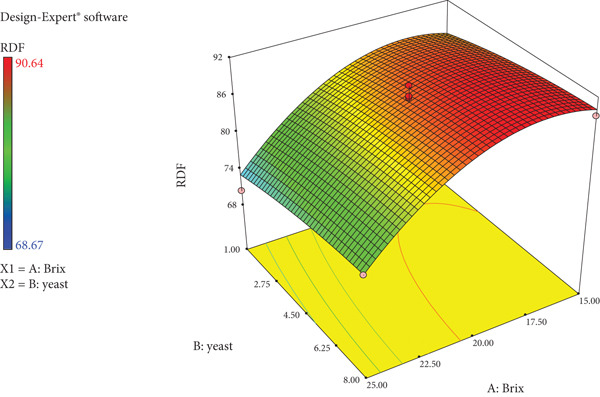
Response surface plot of Brix and yeast on RDF of wild loquat wine. Optimization.

As the sugar concentration (degrees Bx) and yeast amount increase, so do the degrees Plato values of wild loquat wine as seen in Figure 5. A similar linear trend is observed with ABV percentage and calories (Figure 6). The response surface plot of Brix and yeast on RDF of wild loquat wine is noted as Figure 7. The RDF of wild loquat wine gradually increases as the Brix and yeast values increase with a typical concave interaction of variables. Of note is the highest Brix (27.07°Bx) having the lowest noted RDF of 68.67%.

A desirability function was calculated to maximise colour and minimise calories of the optimised product while all the other attributes were kept in range, as shown in Table 5. By applying the desirability function method, two solutions were obtained for the optimum conditions covering criteria with desirability values of 0.636. The optimum values of Brix and yeast for the fermentation of mazhanje wine were found to be 24.44°Bx and 1 g/L, respectively. The predicted values for the responses are 11.83% (ABV), 1.00539 g/cm^3^ (density), 75% (RDF), 5.53 (EBC colour), 350.998 kJ/100 mL or 87 kcal and a pH of 3.5, as shown in Table 6.

**Table 5:** Optimization criteria for the fermentation process of wild loquat wine.

**Name**	**Goal**	**Lower limit**	**Upper limit**
Brix (°Bx)	Is in range	8	25
Yeast (ppm)	Is in range	1	8
ABV (%)	Is in range	8	13
Density (g/cm^3^)	Is in range	0.99	1.04
RDF (%)	Is in range	50	80
Colour (EBC)	Maximise	1.3	6.01
Calories (kJ/100 mL)	Minimise	175.72	402.41
pH	Is in range	3	4

**Table 6:** Numerical optimisation of parameters for alcoholic fermentation.

**Solution**	**Brix (°Bx)**	**Yeast (ppm)**	**ABV (%)**	**Density (g/cm** ^**3**^ **)**	**RDF (%)**	**Colour (EBC)**	**Calories (kJ/100 mL)**	**pH**	**Desirability**	**Remark**
**1**	**24.44**	**1.00**	**11.8307**	**1.00539**	**75.1997**	**5.52553**	**350.998**	**3.49863**	**0.636**	**Selected**
2	24.37	1.00	11.8005	1.00514	75.4304	5.49387	349.841	3.49948	0.636	

*Note:* Selected optimum values are in bold.

### 3.1. Sensory Evaluation

Table 7 shows a summary of the sensory evaluation of the fruit wines. Colour and clarity were the most preferred attributes, while off odour and sourness were the least preferred attributes.

**Table 7:** Summary statistics for sensory evaluation.

**Variable**	**Observations**	**Minimum**	**Maximum**	**Mean**	**Std. deviation**
Colour	13	1.000	5.000	3.615	1.193
Clarity	13	2.000	5.000	4.000	1.225
Fruity aroma	13	1.000	5.000	2.692	1.377
Ethanol aroma	13	1.000	5.000	2.692	1.377
Off odour	13	1.000	5.000	1.769	1.481
Body	13	1.000	5.000	2.846	1.214
Sourness	13	1.000	4.000	2.308	1.182
Sweetness	13	1.000	5.000	2.615	1.502
Aftertaste	13	1.000	5.000	3.538	1.391

**Figure 8:**
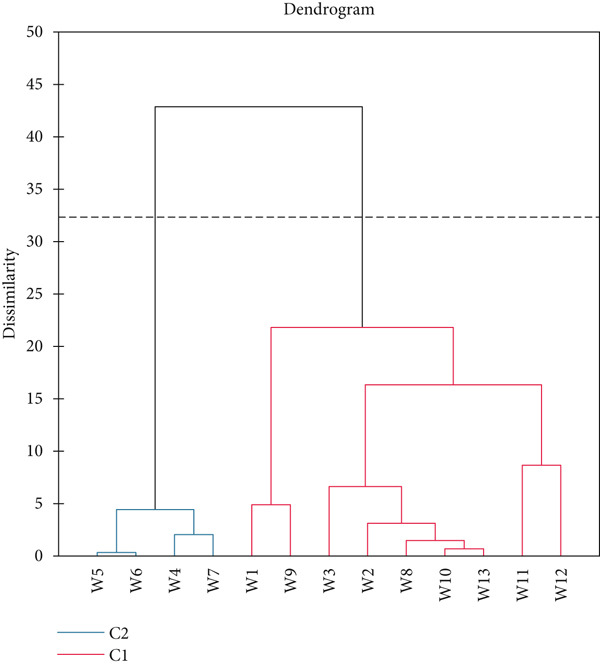
Agglomerative hierarchical clustering (AHC) dendrogram.

**Figure 9:**
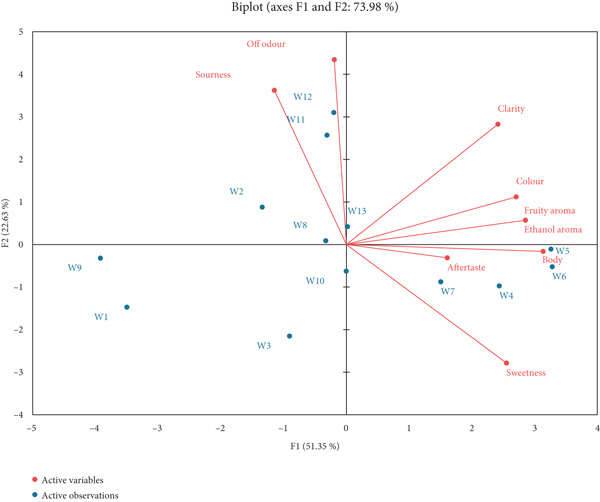
Principal component analysis (PCA) biplot of sensory evaluations by the trained sensory panel (*n* = 13) of the 13 wine samples.

Figure 8 shows wine segmentation (*n* = 13) by cluster analysis as evaluated by the panellists. Agglomerative hierarchical cluster analysis is performed, and the dissimilarity is measured by Euclidean distance and aggregation [26]. The level of truncation can be seen by the dashed line. Cluster 1 included wine samples W5, W6, W4 and W7, whereas Cluster 2 had W1, W9, W3, W2, W8, W10, W13, W11 and W12. The first two components in Figure 9 described 73.98% of the variation in the data. PC 1 displays the separation of the wines primarily on the basis of the attributes ‘clarity’, ‘colour’, ‘fruity aroma’, ‘ethanol aroma’, ‘body’, ‘aftertaste’ and ‘sweetness’ on the right side of the PC compared with ‘sourness’ and ‘off odour’ on the left. PC 2 separated the samples with the appearance attribute ‘colour’, the aroma attributes ‘fruity aroma’ and ‘ethanol aroma’ and also the in‐mouth attribute ‘body’, each of which were strongly positively loaded on PC 2. Wine samples W2, W8, W11, W12 and W13 are located in the top half of Figure 9, while W1, W3, W4, W5, W6, W7, W9 and W10 are located in the bottom section and rated low in the attributes loaded positively on PC 2.

## 4. Discussion

### 4.1. Effect of Brix and Yeast on Responses

*S. cerevisiae* is generally found in association with the production of alcoholic beverages and it is strongly specialised for fermenting high sugar substrates. For its fermenting features and oenological aptitude, *S. cerevisiae* is the species that conducts and determines the rightness of the fermentation process characterising the chemical and sensory profile of wine [27, 28]. The growth of yeasts has been found to be linearly linked with the production of these alcohols, but there are other factors that are responsible for the production of higher alcohols, including fruit, maturation, composition of amino acids, strain of yeast and fermentation conditions, viz., temperature and pH [3]. Therefore, one of the most important parameters monitored for wine production is the sugar content of the starting material, and the higher initial sugar contents could result in higher levels of ethanol after alcoholic fermentation [29]. This is evident in Figure 3 which shows that as the Brix level increased, so does the ABV percentage of the wine. The highest Brix level of 27.07°Bx had an ABV of 11.59%, while the lowest Brix of 12.93°Bx had an ABV of 6.8%. In a related study, total soluble sugars (TSS) decreased from 18°Bx in must to 4.3°Bx in wine [30]. The decrease in total sugar content from must to wine was indicative of the consumption of the sugar sources by the wine yeast to produce ethanol.

The density of the wine is primarily determined by the concentration of alcohol, sugar, glycerol and other dissolved solids. It is known that increasing the alcohol content decreases density [31] and alcohol enhances the sensory descriptor of body and fullness in wine [32]. Nurgel and Pickering [33], in their study on the contribution of glycerol, ethanol and sugar to the perception of viscosity and density of model wine solutions, found that across a range of concentrations investigated, sugar influenced the perception of viscosity and density the most. However, in this study, the density value was 0.9939 g/cm^3^ when Brix was 12.93°Bx (6.8 ABV %) and the highest density recorded was 1.01239 g/cm^3^ at 27.07°Bx and 11.59 ABV % (Table 2) indicating the influence of other metabolites other than sugar content. Density of red wine is expected to be between 0.990 and 1.004 g/cm^3^, with that of white wine being expected to be from 0.987 to 1.039 g/cm^3^ [34]. This shows that the results obtained in this study are within the range of expected densities of wine.

RDF measures the degree to which sugar in wort or must has been fermented into alcohol in beer, cider or wine, also defined as attenuation [35]. The RDF expresses the percentage of extract that was fermented. RDFs in the 50s represent with over 40% of their original extract left unfermented, whereas RDFs in the 80s represent highly attenuated beverages with less than 20% of their original extract unfermented. The RDF values obtained in this study ranged from 68.67% to 90.64%, indicating high attenuation. There was a strong negative correlation between Brix and RDF (*r* = –0.727), while there was a weak positive correlation (*r* = +0.201) between yeast and RDF, which is in line with literature that attributes the degree of fermentation to the activity of yeast [36, 37].

The calorie values of the wine ranged from 175.72 (43.75 kcal) to 402.41 kJ/100 mL (100.5 kcal), with a strong positive correlation (*r* = +0.950) between calories and initial Brix, as seen in Table 4. Figure 6, which showed the correlation between ABV percentage and calories of wild loquat wine to be a strong positive (*r* = +0.927), can be attributed to alcohol’s high calorie per gramme (7 kcal/g) [38]. The correlation between Brix and calories can be explained by the fact that sucrose, as a pure carbohydrate, has an energy content of 4 kcal/g [39]. Therefore, the higher the degrees Brix, the higher the calories of the wine. Fruit wines contain 8–11 ABV % and 2%–3% (*w*/*v*) sugar, with an energy value ranging between 70 and 90 kcal/100 mL [40]. The standard 175 mL glass of 12% wine contains 133 kcal, while 100 mL of 17.5% fortified wine contains 154 kcal [38]. This indicates that the wild loquat wine produced in this study has calories within the expected range. There was a strong positive correlation between colour and Brix (*r* = +0.682) and a weak positive correlation between colour and yeast (*r* = +0.210) indicating that both factors influenced the factor. The wine colour was measured on an EBC scale, which was developed as a way to standardise measuring on beer colour, a process that can be subjective rather than technical [35]. The wine’s colour was found to range from 1.30 to 6.01 on the EBC scale. In wild loquat wine, phytochemicals contribute to colour. Phenolic compounds such as flavonoids (anthocyanins, flavanols, flavonols, etc.), betalains (betaxanthins and betacyanins), organic acids and nonflavonoids (derived from cinnamic and benzoic acids), which have efficient antioxidant, anticancer and antimicrobial activities, are present in fruit wines [41], and these may contribute to colour.

During fermentation, the pH of the wine reaches a value of 3.5–3.8, suggesting that an acidic fermentation takes place at the same time as the alcoholic fermentation [8]. In the present study, wine pH ranged in values from 3.48 to 4.35, corresponding with values of other fruit wines such as cactus pear and *Lantana camara* wine (pH = 3.47), watermelon wine (pH = 4.00), pawpaw (pH = 3.4) and a range of 3.6–4.3 in blended fruit wine containing banana, watermelon and pawpaw as well as sapota fruit wine (pH = 4.45) [41–43]. A pH of 3.4 inhibits spoilage bacteria, and wine yeast can metabolise at this pH level [24]. The correlation shown in Table 4 indicated that when the Brix values were increasing, the pH values decreased (*r* = –0.211), signifying higher metabolic activities during fermentation to produce organic acids, hence the decrease in pH. The perception of acidity in wine is reduced with increasing alcohol content. Consequently, wines with reduced alcohol content appear more acidic [32]. The pH also plays an important role in ageing, clarifying or fining. As the strength of the relative charge of suspended particles decreases in the wine, the pH of the wine increases. At high pH, organic protein fining agents may possess a positive charge insufficient to bind to the negatively charged particulates, thus potentially increasing the turbidity of the wine [8].

### 4.2. Sensory Evaluation

Sensory profiles are used to identify the main qualitative and quantitative sensory dimensions of products [44]. The sensory properties of fruit wines are widely discussed elsewhere by Zhu et al. [45]. PCA is a technique for reducing the dimensionality of such datasets, increasing interpretability but at the same time minimising information loss [46]. In this study, sensory aspects of the finished wild loquat wine samples were assessed in relation to initial Brix and yeast. Figure 8 shows that wine samples W5 and W6 were most similar, followed by samples W10 and W13. While W10 and W13 had the exact same initial Brix and yeast, W5 and W6 had both factors being different initially. Agglomerative hierarchical cluster analysis has provided useful information in wine production [47, 48].

To investigate the role aromatic substances play in the quality of wine, PCA can be a useful tool. Wine yeasts produce metabolites known to influence sensory characteristics of wine, for example, higher alcohols, esters, volatile acids, carbonyl compounds, volatile phenols and sulphur compounds [2]. The intensities of colour, clarity, fruity aroma, ethanol aroma, off odours, body, sourness, sweetness and aftertaste were evaluated for all 13 wines. Table 7 showed that colour and clarity were the most preferred attributes. The proximity of the attributes to a specific wine sample in Figure 9 reflected its degree of association. Samples W9, W1, W3 and W10 were not significantly linked to any of the attributes that the panellists were assessing. This indicated that the samples were of low intensity when it came to all the attributes that were being assessed. Wine sample W1 and W9 had the lowest initial Brix, which could be attributed to this weakness. Samples W11 and W12 had the highest intensities when it came to sourness and off odour, with a number of panellist commenting that the samples were their least favourites overall. The off odour (any unpleasant or unexpected smell) may have been caused by stuck or sluggish fermentations [49]. The fermentation and maturation process of fruit wine can increase the release of flavonoid aglycones that are more active phytochemicals than their conjugated forms [2]. From a technological standpoint, phenolic compounds are key determinants of several organoleptic attributes of fruit wines, including colour, taste, astringency and bitterness [3]. Phenolic acid is composed mainly of chlorogenic and p‐coumaroylquinic acid, together with dihydrochalcones such as phloridzin and phloretin 2‐xyloglucosides and flavonols, which are related to bitterness [3]. Sample W2 (initial yeast amount at 0.45 g/L), sample W11 (initial yeast amount at 9.45 g/L) and W12 (initial yeast amount at 1 g/L) were closely linked to the two attributes (sourness and off odour). This could be attributed to low acidity. Low acidity reduces flavour harmony, while high acidity raises the sense of sourness. Titratable acidity has a direct influence on the aroma and flavour of fermented products, and the amount present could be an indicator of shelf life [11]. Samples W5 and W6 also had the most notable fruity and ethanol flavour. This could be attributed to secondary metabolites. Esters, higher alcohols, acetates, organic acids and other compounds are the groups of volatile compounds that most commonly contribute to the flavour and/or aroma profile of fruit [2]. These metabolites also contribute to fruit colour and positively influence specific health attributes of fruits such as wild loquat. The association between yeast and colour is weak as shown in Table 4. Fruits contain various dietary phytonutrients with strong antioxidant capacities, such as phenolics and vitamins. Phenolic compounds, namely, phenolic acids, anthocyanins, flavonols, catechins and other flavonoids present in fruit wines, significantly influence the wine quality since they have an impact on the sensory characteristics of wines, mainly astringency and colour [50]. While yeast has an influence on flavour of the fruit wine, its impact on colour is minimal. The ageing of the wine will also modify the phenolic composition because the phenolic compounds undergo different transformations, such as oxidation, condensation and polymerisation reactions. All these reactions are related to the colour. However, ageing process in wines is not influenced by yeast.

## 5. Conclusion

Wild loquat fruits are an underutilised, nutritious fruit with the potential to be commercially processed into a fruit wine. The results suggested that RSM by CCD can be used to navigate the design space for optimisation of alcoholic fermentation of wild loquat wine. The optimal parameters for wild loquat wine fermentation were found to be 24.44°Bx and 1 ppm for Brix and yeast concentration, respectively. The study showed that colour and clarity were the most preferred attributes. Off odours and sourness were intense in wine samples with extreme initial values for yeast. Optimisation of the fermentation time and temperature of wild loquat wine using the optimised values for Brix and yeast from this study is recommended.

## Conflicts of Interest

The authors declare no conflicts of interest.

## Author Contributions

All the authors were involved in the conceptualisation, search, writing and editing of the paper.

## Funding

No funding was received for this manuscript.

## Data Availability

Data will be made available upon request.
